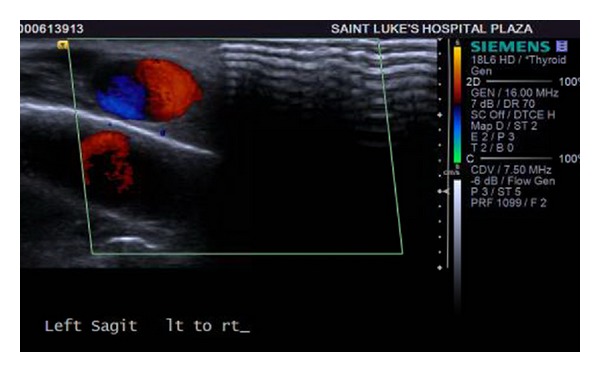# Novel Onset of a Posttraumatic Superficial Temporal Artery Pseudoaneurysm

**DOI:** 10.1155/2013/369309

**Published:** 2013-07-17

**Authors:** Alexandra A. Roman, Andre J. Arsenault, Kenneth D. Jackson, John M. Price

**Affiliations:** Department of Surgery, St. Luke's Hospital, 4320 Wornall Road Medical Plaza 1, Suite 530, Kansas City, MO 64111, USA

## Abstract

Less than 200 cases of posttraumatic superficial temporal artery pseudoaneurysm have been described in the literature. The majority of these cases result from blunt head trauma and are diagnosed an average of three weeks following the inciting traumatic event. In this case report, we describe a superficial temporal artery pseudoaneurysm that developed and was diagnosed the same day of a blunt head trauma in a 54-year-old white male. This is the earliest formation/diagnosis of post-traumatic superficial temporal artery pseudoaneurysm yet reported in the literature. This case report demonstrates that this diagnosis should be kept in the list of differential diagnoses for a post-traumatic soft tissue mass of the face, even immediately following the traumatic event.

## 1. Introduction

Posttraumatic aneurysms do not commonly arise within the distribution of the facial artery, as the external carotid and its branches are protected in most locations by overlaying soft tissue. When they do occur, one of the most commonly affected vessels is the anterior branch of the superficial temporal artery (STA) because of its superficial course over the frontal bone, between the frontalis and temporalis muscles [1]. The majority (89%) of traumatic STA aneurysms are actually pseudoaneurysmal and form when partial transection of the arterial wall leads to extravasation of blood that gradually displaces surrounding soft tissues until they form a fibrous pseudocapsule enclosing a hematoma [2]. Fewer than 200 cases of traumatic STA pseudoaneurysm have been reported in the literature [2]. Given their potential for rupture and hemorrhage, it is necessary to differentiate STA pseudoaneurysms from more common diagnoses so that subsequent complications can be prevented. 

## 2. Case Report

A 54-year-old Caucasian male with a past medical history significant for anaplastic astrocytoma previously treated with chemoradiation was examined in the emergency department after experiencing two seizure-like episodes earlier that morning. These episodes involved rhythmic movements of the right arm, confusion, and aphasia. During the second episode, which occurred in the parking lot of the emergency department, the patient fell and repeatedly impacted the left side of his face on the pavement. Upon examination, the patient had significant abrasions to the left side of the face with a small, palpable subcutaneous mass over the left temporal region. The patient demonstrated no focal neurological deficits. A computed tomography (CT) scan of the patient's head obtained in the emergency department demonstrated a small focus of intraparenchymal hemorrhage for which the patient was admitted to the hospital for further observation and management.

**Figure 1:**
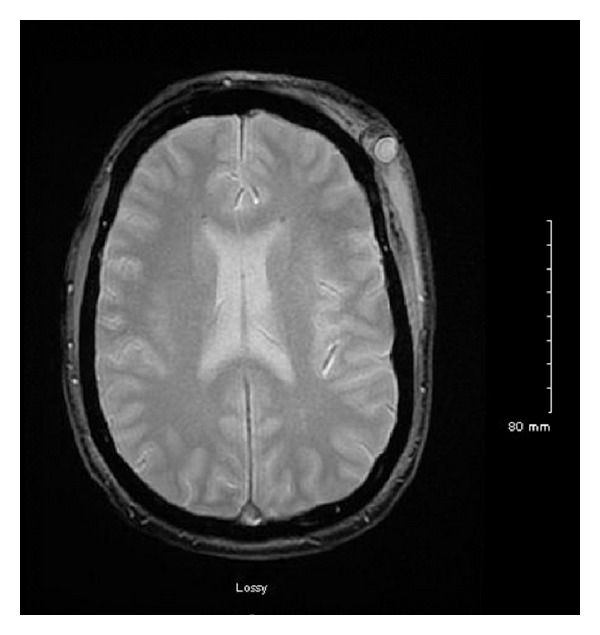


**Figure 2:**
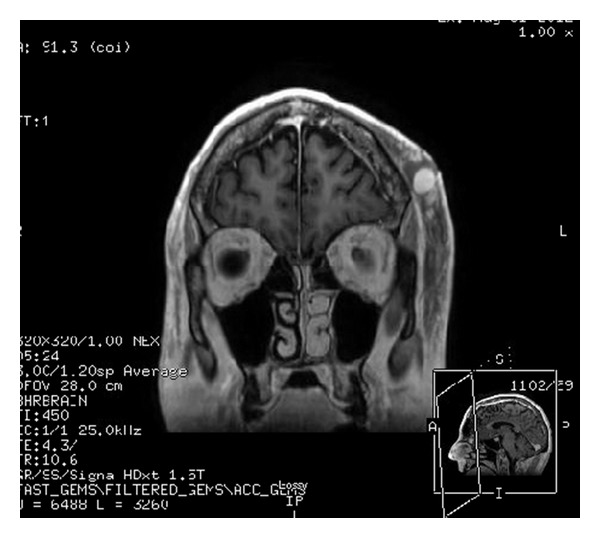


**Figure 3:**
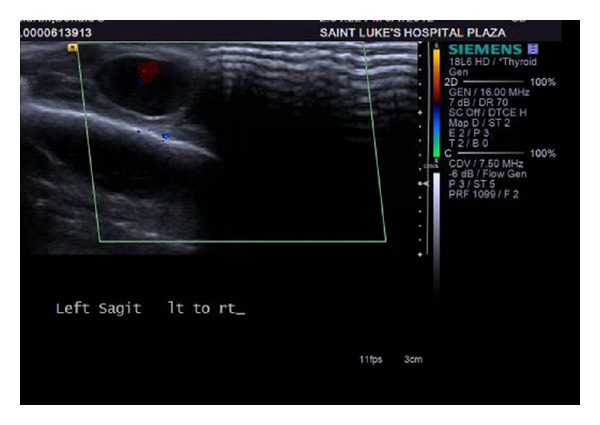


**Figure 4:**
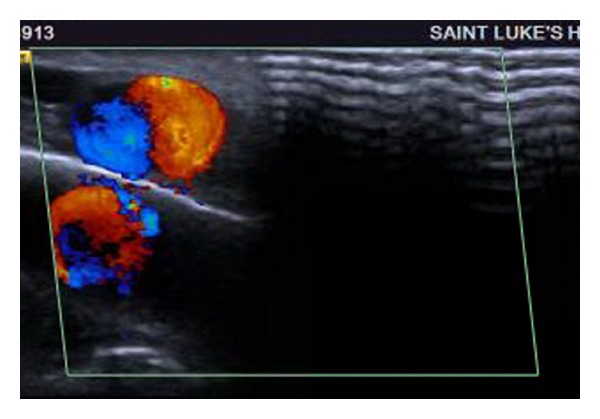


The following day, a clinical examination revealed that the small bulge over the left temporal region had doubled in size (1 cm × 1 cm), was pulsatile, and freely mobile with no obvious involvement of the overlying skin. No bruit or thrill was appreciated. The patient reported minimal pain associated with the mass. A magnetic resonance image (MRI) was obtained at the suggestion of the consulting neurologist that redemonstrated a focal hemorrhage in the vertex of the left cerebral hemisphere with surrounding edema (Figures 1 and 2). The MRI also showed a 1.3 cm × 1.4 cm circumscribed, enhancing lesion in the soft tissue of the left scalp, with an appearance consistent with that of a posttraumatic pseudoaneurysm. Doppler ultrasound of the left scalp mass revealed a 1.0 cm × 1.3 cm circular lesion in the soft tissue of the left anterior temporal scalp with bidirectional flow, consistent with a pseudoaneurysm (Figures 3, 4, and 5). The patient was subsequently discharged on seizure prophylaxis in stable condition with no pain or neurologic deficit. Four days following discharge, the patient represented with complaints of increasing pain in the area of the pseudoaneurysm. On focused physical exam, the left temporal bulge was noted to have increased in size (now several centimeters across), and the formation of a central black eschar overlying the mass was noted. The mass was pulsatile and tender to palpation. 

 The patient underwent surgical excision of the pseudoaneurysm under general anesthesia. The lesion was approached through an elliptical incision overlying the pseudoaneurysm, which included the skin and subcutaneous tissue down to the level of the pseudoaneurysm. Upon entering the pseudocapsule, there was a mild amount of oozing and a large hematoma and clot, which were all evacuated. The proximal and distal ends of the STA that had been feeding the pseudoaneurysm were identified and ligated. The wound was closed with vertical mattress sutures after hemostasis was verified. The patient's postoperative course was uneventful.

## 3. Discussion

Of the cases of traumatic STA pseudoaneurysm that have been reported in the literature, 95% have resulted from blunt trauma, while the remaining cases resulted from penetrating trauma or iatrogenic cause [3]. The most common types of injury associated with the development of post-traumatic STA pseudoaneurysm include assault, falls, sports related, and iatrogenic causes including excision of basal cell carcinoma, hair transplantation, and CT-guided biopsies [2].

 Patients typically present an average of three weeks following the inciting traumatic event. However, a literature review of STA pseudoaneurysms reported a range of two days to 17 years from trauma to diagnosis [2]. Patients most commonly present with an enlarging painless, pulsatile cystic mass with or without a throbbing headache; in rare cases, patients have reported pain in the area of the mass or visual and auditory disturbances. Clinical examination typically reveals a pulsatile mass in the frontotemporal region. In some cases, pulsation may be absent if there is complete thrombosis of the aneurysmal sac. This can mimic more common diagnoses including sebaceous cysts, lipomas, arteriovenous fistulas, simple hematomas, inflamed lymph nodes, and neuromas of the supraorbital nerve [1, 4, 5]. Compression of the artery proximal to the mass should minimize pulsatility. In a few reported cases, a thrill or bruit was noted [6].

 In a minority of cases of posttraumatic STA pseudoaneurysms, history of a head injury in combination with a focused physical exam was sufficient to make the diagnosis [1–6]. If imaging is necessary to confirm the diagnosis, the most successful noninvasive modality is a Duplex Doppler ultrasound [6, 7]. CT and MRI may be useful to see associated intracranial trauma, but are not suggested for first-line imaging [2]. The definitive diagnosis can be made with angiography, but, as this is an invasive test it, is should be reserved for diagnosis of difficult cases [1, 6, 8, 9]. While physical exam in combination with imaging is recommended, it should be noted that needle aspiration of the lesion as a diagnostic tool should be avoided due to the risk of bleeding, which could prove difficult to control in an outpatient setting.

 Given the low incidence of STA pseudoaneurysm, no guidelines exist to direct management. However, given the risk of thromboembolism, spontaneous rupture, and hemorrhage in addition to the associated pain and cosmetic defect, it is necessary that STA pseudoaneurysms are accurately identified and treated [1, 2]. Treatment modalities range from conservative observation to curative endovascular treatments or surgical ligation and resection. While there have been many advances in minimally invasive treatments including direct percutaneous thrombin injection and endovascular coil embolization [10, 11], surgical resection remains the gold standard treatment and is associated with the lowest rate of recurrence and postoperative complications [2].

 To our knowledge, the present case is the first report in the literature of a post-traumatic STA pseudoaneurysm that was present the day of the inciting trauma. On average, STA pseudoaneurysms due to trauma develop approximately three weeks following a traumatic incident, with the earliest report in the literature being two days after-trauma [12]. This case report illustrates the need to maintain the diagnosis of STA pseudoaneurysm in the differential, even immediately following a traumatic event, despite the uncharacteristic presentation of this particular pathologic entity.

## Figures and Tables

**Figure 5:**